# The Untouchable Ventral Nucleus of the Trapezoid Body: Preservation of a Nucleus in an Animal Model of Autism Spectrum Disorder

**DOI:** 10.3389/fnint.2021.730439

**Published:** 2021-09-29

**Authors:** Yusra Mansour, Randy J. Kulesza

**Affiliations:** ^1^Department of Otolaryngology, Henry Ford Macomb Hospital, Clinton Township, MI, United States; ^2^Department of Anatomy, Lake Erie College of Osteopathic Medicine, Erie, PA, United States

**Keywords:** autism, hearing – disorders, brainstem, olivocochlear, valproate

## Abstract

Autism spectrum disorder (ASD) is a neurodevelopmental condition characterized by repetitive behaviors, poor social skills, and difficulties with communication and hearing. The hearing deficits in ASD range from deafness to extreme sensitivity to routine environmental sounds. Previous research from our lab has shown drastic hypoplasia in the superior olivary complex (SOC) in both human cases of ASD and in an animal model of autism. However, in our study of the human SOC, we failed to find any changes in the total number of neurons in the ventral nucleus of the trapezoid body (VNTB) or any changes in cell body size or shape. Similarly, in animals prenatally exposed to the antiepileptic valproic acid (VPA), we failed to find any changes in the total number, size or shape of VNTB neurons. Based on these findings, we hypothesized that the neurotransmitter profiles, ascending and descending axonal projections of the VNTB are also preserved in these neurodevelopmental conditions. We investigated this hypothesis using a combination of immunohistochemistry and retrograde tract tracing. We found no difference between control and VPA-exposed animals in the number of VNTB neurons immunoreactive for choline acetyltransferase (ChAT). Additionally, we investigated the ascending projections from the VNTB to both the central nucleus of the inferior colliculus (CNIC) and medial geniculate (MG) and descending projections to the cochlea. Our results indicate no significant differences in the ascending and descending projections from the VNTB between control and VPA-exposed animals despite drastic changes in these projections from surrounding nuclei. These findings provide evidence that certain neuronal populations and circuits may be protected against the effects of neurodevelopmental disorders.

## Introduction

The ventral nucleus of the trapezoid body (VNTB) is one of the periolivary nuclei within the superior olivary complex (SOC) – a multichannel processing station along the mammalian auditory pathway. VNTB neurons reside within the decussating axons of the trapezoid body that originate from neurons in the ventral cochlear nucleus (VCN) and are directed largely toward the SOC and nuclei of the lateral lemniscus. The VNTB includes about 4,500 neurons in rat (Kulesza et al., 2002) and 1,400 neurons in human (Kulesza, 2008). The VNTB includes a number of distinct neurochemical populations. There are populations of both large and small cholinergic neurons (see below; Warr, 1975; Sherriff and Henderson, 1994; Warr and Beck, 1996). There are also glycinergic (Saint Marie and Baker, 1990) and GABAergic populations (Roberts and Ribak, 1987; Albrecht et al., 2014) and a population that likely co-localizes these neurotransmitters (Albrecht et al., 2014). In fact, during the early postnatal period VNTB neurons transition from using gamma amino butyric acid (GABA) to glycine as a neurotransmitter (Albrecht et al., 2014).

The VNTB receives ascending input from globular bushy cells, octopus cells, and multipolar cells in the contralateral (CL) VCN (Warr, 1972; Friauf and Ostwald, 1988; Kuwabara and Zook, 1991; Smith et al., 1991; Thompson, 1998) and smaller projections from the ipsilateral (IL) VCN. The VNTB also receives input from the IL medial nucleus of the trapezoid body (MNTB; Kuwabara and Zook, 1991). There is also a descending projection from the IL central nucleus of the inferior colliculus (CNIC; Caicedo and Herbert, 1993; Vetter et al., 1993). Consistent with such a wide range of inputs, the VNTB projects extensively throughout the auditory brainstem. The best characterized projection is part of the olivocochlear (OC) system that projections *via* the olivocochlear bundle (OCB) to outer hair cells in the cochlea; this is a bilateral projection with a contralateral (CL) predominance (rat – White and Warr, 1983; cat – Warr et al., 2002). This vast majority of VNTB neurons projecting to the cochlear nucleus (CN) and cochlea are cholinergic (Dannhof et al., 1991; Vetter et al., 1991). While OC neurons in the VNTB may send collateral projections to the CN, there are some smaller neurons that are choline acetyltransferase positive (ChAT+) neurons that project to the dorsal and ventral cochlear nuclei and cochlear root neurons *via* the trapezoid body (Osen et al., 1984; Godfrey et al., 1987; Benson and Brown, 1990; Sherriff and Henderson, 1994; Warr and Beck, 1996; Gómez-Nieto et al., 2008). The VNTB makes a glycinergic projection *via* the lateral lemniscus to the IL inferior colliculus (Saint Marie and Baker, 1990; Warr and Beck, 1996). Finally, there are local projections within the SOC to the MNTB, lateral nucleus of the trapezoid body (LNTB) and lateral superior olive (LSO; Warr and Beck, 1996; Albrecht et al., 2014). Based on these observations, the VNTB is a heterogeneous nucleus that receives both ascending and descending inputs. It forms a major component of the medial olivocochlear system that modulates the sensitivity of the organ of Corti and projects to cochlear root neurons to influence the acoustic startle reflex. The VNTB projects locally within the SOC and along the ascending auditory pathway where it functions in sound localization and coding spectral and temporal features of sound. Indeed, the VNTB is situated to function in a number of important aspects of brainstem auditory processing.

Auditory processing deficits are common in subjects with autism spectrum disorders (ASD) and in animal models of ASD (Greenspan and Wieder, 1997; Tomchek and Dunn, 2007; Gomes et al., 2008; Bolton et al., 2012; Danesh and Kaf, 2012; O’Connor, 2012). In fact, human subjects with ASD have auditory brainstem responses (ABR) and stapedial reflexes with longer latency, decreased amplitude, and right-left asymmetry (Skoff et al., 1980; Rumsey et al., 1984; McClelland et al., 1992; Klin, 1993; Kwon et al., 2007; Roth et al., 2012; Lukose et al., 2013). *In utero* exposure to the antiepileptic valproic acid (VPA) results in increased risk of an ASD diagnosis in humans and is a validated animal model of ASD (Rodier et al., 1996; Moore et al., 2000; Williams et al., 2001; Rasalam et al., 2005; Koren et al., 2006; Bromley et al., 2013; Christensen et al., 2013; Mabunga et al., 2015).

Our previous research has revealed significantly fewer neurons in the SOC of both human subjects (ranging in age from 2 to 52 years of age) diagnosed with ASD and in VPA-exposed animals (Kulesza and Mangunay, 2008; Kulesza et al., 2011; Lukose et al., 2015). In fact, in both human cases of ASD and VPA-exposed animals, we found significantly fewer neurons in the medial superior olive (MSO), LSO, MNTB, LNTB, and superior paraolivary nucleus (SPON; Kulesza et al., 2011; Lukose et al., 2015). However, morphology of VNTB neurons was not significantly different in our study of over 56 human subjects with ASD and in our study of VPA-exposed animals (Kulesza and Mangunay, 2008; Kulesza et al., 2011; Lukose et al., 2015; Zimmerman et al., 2018). Specifically, we found no differences in the total number of neurons and no differences in the size or shape of VNTB neurons, even when split by cell type (Kulesza et al., 2011; Lukose et al., 2015; Zimmerman et al., 2018). These observations led us to consider that despite the drastic changes in the surrounding SOC nuclei, the VNTB is spared in ASD and animal models of this condition. Further, 3D volumetric models of the human SOC revealed all SOC nuclei were significantly smaller, except for the VNTB (Mansour and Kulesza, 2020).

These observations led us to hypothesize that VPA exposure does not impact ascending or descending projections or neurotransmitter profiles in the VNTB. To examine this hypothesis, we undertook retrograde tract tracing experiments using Fluorogold (FG) or Fast Blue (FB). We examined ascending projections to the medial geniculate body (MG), or CNIC and examined descending projections to the cochlea by injections of FG at the round window. We finally correlated retrogradely labeled neurons with neurotransmitter profile in double-labeling experiments for choline acetyltransferase (ChAT).

## Materials and Methods

### Valproic Acid Exposure

**FIGURE 1:**
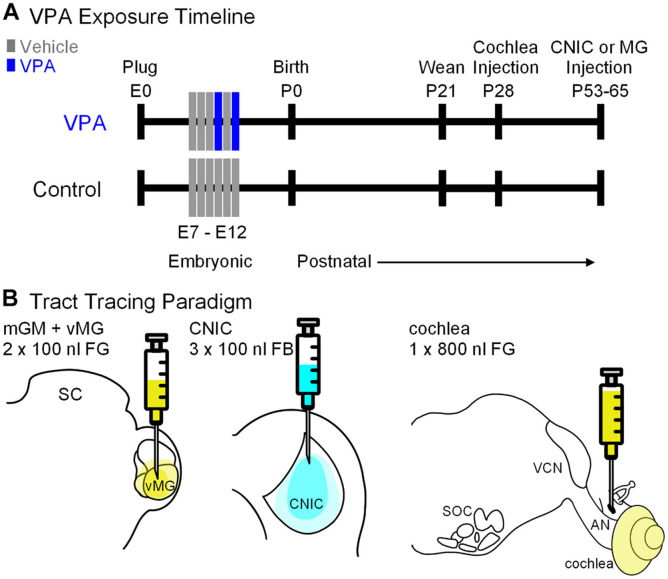
Study design. Shown in **(A)** is the timeline for the study. Pregnant females were fed peanut butter on E7–12 and animals in the VPA group received peanut butter with VPA on E10 and 12. Pups were weaned on P21. Cochlear injections were made on P28. Injections into the CNIC or MG were made between P53–65. Shown in **(B)** is the tracer and volume injected into each target.

All handling and surgical procedures were approved by the LECOM Institutional Animal Care and Use Committee (protocols #16-02, 18-03, 19-04, and 20-02) and conducted in accordance with the National Institute of Health Guide for the Care and Use of Laboratory Animals. Sprague–Dawley rats were maintained on a 12 h light/dark cycle with *ad libitum* access to food and water. *In utero* exposure to VPA was performed as previously described (Figure 1A; Main and Kulesza, 2017; Zimmerman et al., 2018, 2020; Mansour et al., 2019, 2021). Briefly, dams were fed 3.1 g of peanut butter on embryonic days (E) 7–12. On E10 and E12, dams in the VPA group were fed peanut butter mixed with 800 mg/kg of VPA (Figure 1A). Control animals were fed peanut butter meals without VPA. Both control and VPA-exposed dams were permitted to deliver pups without interference (litters were not culled). On postnatal day (P) 21, litters were weaned and only male pups were included in the study since gender-specific effects of VPA exposure are established (Schneider et al., 2008). We conducted this study under the assumption that all male pups in a given litter were equally affected by VPA exposure; our previous studies provide data consistent with this strategy (Main and Kulesza, 2017; Zimmerman et al., 2018, 2020; Mansour et al., 2019, 2021). We additionally utilized archival collections of Giemsa-stained tissue sections (i.e., every 3rd tissue section at a thickness of 40 μm) from previous investigations as reference for morphological features of the VNTB (Zimmerman et al., 2018; Mansour et al., 2019). While our previous work provides evidence for abnormal tonotopic maps and/or hyperactivation of brainstem centers in VPA-exposed animals (Dubiel and Kulesza, 2016) and abnormal ascending projections to the midbrain and thalamus (Zimmerman et al., 2020; Mansour et al., 2021), we did not perform any hearing tests or audiometric screening on the animals used in this study.

### Surgery and Tracer Injections

Cochlear injections were made on P28 (Figure 1B). Animals were placed in an induction chamber and anesthetized with vaporized isoflurane (5% induction, 2.5–3% maintenance, O_2_: 1.2 l/min). Once animals were unresponsive, they were removed from the chamber, fit with a custom face mask providing continuous anesthesia and secured in a custom foam support. Body temperature was maintained *via* a heating pad. The scalp was disinfected with 70% ethanol and washed with iodine solution. A retroauricular approach was taken to the cochlea. After cutting through the skin, blunt dissection was used to reach the bulla; the bulla was opened along the caudal aspect sufficient to visualize the stapedial artery and round window. A 1 μl Hamilton syringe (32 gauge and 4 point) was used to inject 800 nl of 4% FG (Fluorochrome). We did not observe any abnormalities of the auditory bulla, cochlear promontory or oval window in VPA-exposed animals. After the injection, the wound was closed and the animal was returned to their home cage and permitted to recover for 6 days. Injections into the CNIC or MG nuclei were made between P50 and P63 *via* stereotaxic craniotomy as previously described (Figure 1; Zimmerman et al., 2020; Mansour et al., 2021). Animals receiving these injections were anesthetized as above but were secured in a stereotaxic frame with non-rupture ear bars (Kopf Instruments). A midline incision was made in the scalp to expose the dorsal aspect of the skull. The coordinates for CNIC injections were: 0.2 mm rostral to lambda (as indicated by Paxinos and Watson, 2007), 1.5 mm right of the midline. Injections of FB (2.5% in water; Polysciences, Inc.) were made into the CNIC using a 1 μl Hamilton KH Neuros syringe (32 gauge and 4 point; Figure 1B). A depth measurement was taken from the surface of the dura mater and deposits of 100 nl of FB were made at depths of −3.6, −3.2, and −2.6 mm for a total injected volume of 300 nl. The coordinates for MG injections were: 5.6 mm caudal to bregma and 3.4 mm right of the midline (as indicated by Paxinos and Watson, 2007). Injections of FG (4.0% in saline; Fluorochrome) were made using a 1 μl Hamilton KH Neuros syringe (32 gauge and 4 point; Figure 1B). A depth measurement was taken from the surface of the dura mater and deposits of 100 nl of FG were made at depths of −5.8 and −5.0 mm for a total injected volume of 200 nl. After the final injection, the needle was left in place for 10 min. The needle was removed, the bony defect was filled with dental wax and the incision sutured. The wound was injected with lidocaine and the animal taken off isoflurane, returned to their homecage, and monitored until they were able to stand on all fours.

For this study, a stereotaxic injection of FB was made into the CNIC of 10 control animals (from 4 L) and 6 VPA-exposed animals (from 4 L), a stereotaxic injection of FG was made into the MG of 6 control animals (from 6 L) and 5 VPA-exposed animals (from 4 L), and an injection of FG was made into the cochlea of 4 control animals (from 4 L) and 4 VPA-exposed animals (from 4 L). Each animal received only a single injection; we did not attempt any double retrograde labeling experiments.

### Perfusion and Sectioning

Six days following tracer injections, animals were anesthetized with isoflurane and perfused through the ascending aorta with saline followed by 4% paraformaldehyde (PFA) in 0.1 M sodium phosphate buffer (PB). Brains were removed from the skull and the right side was marked with a register pin. Accordingly, the right side is ipsilateral (IL) to the injection and the left side is contralateral (CL). Brains were postfixed in 4% PFA and placed in cryoprotectant (30% sucrose in 4% PFA) 24 h before frozen sectioning. Brains were sectioned in the coronal plane at 50 μm and collected into three wells. Sections from well 1 were archived. Sections from well 2 were used to reconstruct CNIC or MG injection sites. For counting of FB and FG+ neurons, all sections from well 3 were counterstained with Neurotrace Red (a fluorescent Nissl stain; NTR, Invitrogen) and/or processed for immunohistochemistry (see next).

### Immunohistochemistry

Free-floating sections were rinsed in phosphate buffered saline (PBS), blocked in 1% normal horse serum (NHS; Abcam), 0.5% triton X in PBS for 1 h. Sections processed for ChAT were incubated in primary antisera (rabbit anti-ChAT, 1:1000 with 1% NHS; Abcam, catalog #: ab178850) overnight, rinsed in PBS and incubated for 2 h in goat anti-rabbit Dylight 488 (1:100; Vector Labs). Sections processed for glutamate decarboxylase (GAD) were incubated in primary antisera (mouse anti-GAD, 1:250 with 1% NHS; Abcam, catalog #: ab26116) overnight. These sections were then incubated with biotinylated Gt anti-mouse (1:100, Vector Labs) for at least 6 h and then incubated overnight with Streptavidin Dylight 488 (Vector Labs). After the final antibody step, tissue sections were rinsed, and counterstained with Neurotrace Red (Thermo Fisher Scientific), mounted onto glass slides, dried and coverslipped with Entellan (Millipore Sigma).

### Quantification

**FIGURE 2:**
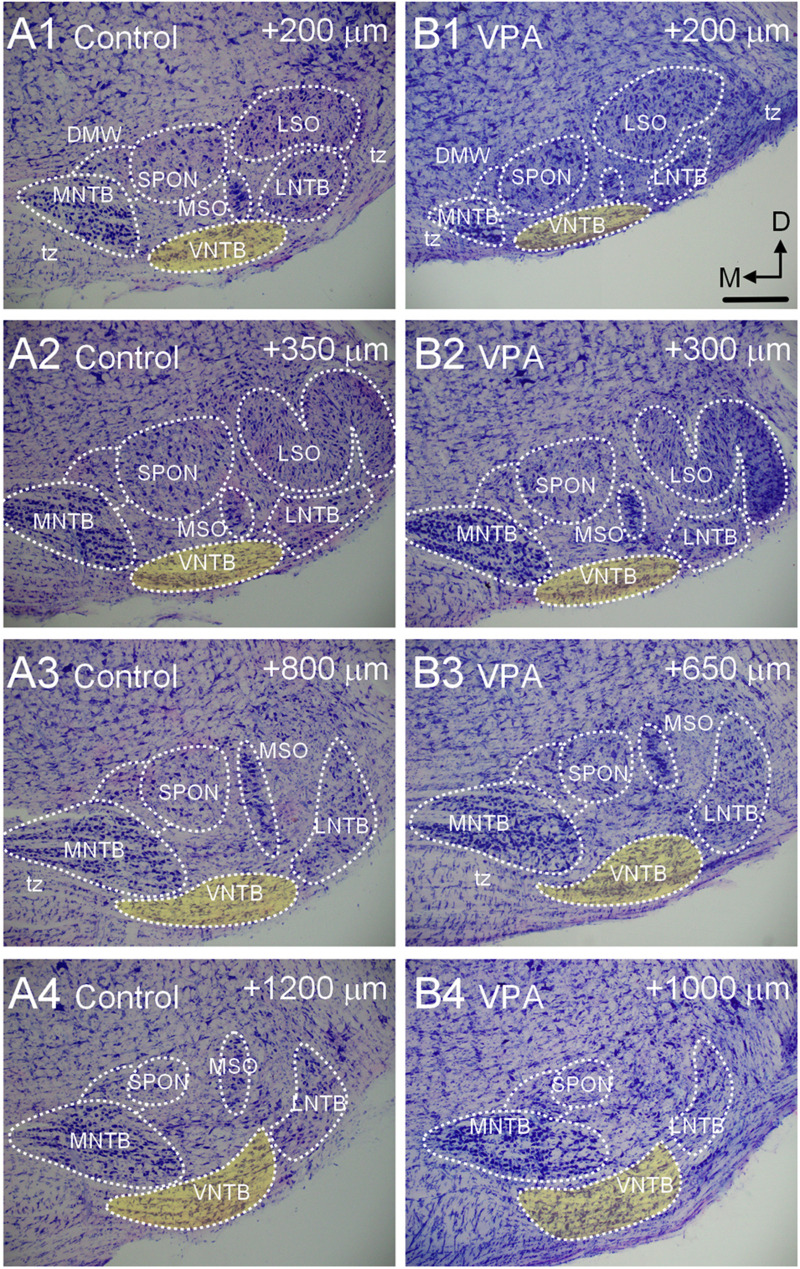
Morphology of the VNTB. Shown in **(A1–4)** is a caudal to rostral series of Giemsa-stained sections through the SOC of a P28 control animal. The VNTB is indicated in yellow. The numbers in the upper right corner of each image indicates how far the section is rostral to the beginning of the SOC in the series. Shown in **(B1–4)** is a similar series through the SOC of a VPA-exposed animal. The SOC in VPA-exposed animals is shorter in the rostrocaudal dimension and besides the VNTB, other nuclei are smaller. The scale bar in **(B1)** is equal to 300 μm.

Injection sites were confirmed and quantified as previously described (Zimmerman et al., 2020; Mansour et al., 2021). Injection sites in the CNIC are the same as published in figures 2 and 3 in Zimmerman et al. (2020) and injection sites in the MG are the same as published in figure 5 in Mansour et al. (2021). Nuclear boundaries of the VNTB were as per previous work on the rat SOC (Kulesza et al., 2002). Photomicrographs were taken with a DP71 digital camera on an Olympus CKX41 microscope or a Leica TCS SP5 confocal microscope. Depending on the experiment, we took photographs of NTR, FG/FB, and ChAT/GAD. Images were overlaid using the stack and z project features in ImageJ (Schindelin et al., 2012). Counts of FG and NTR labeled VNTB neurons were made in at least 3 tissue sections per animal, per CNIC or MG injection. We counted the total number of NTR, FB/FG and ChAT/GAD-labeled neuronal profiles (i.e., triple labeled neurons) in at least two randomly selected sections per animal. Our labeling paradigms revealed no obvious gradients of FG labeled neurons from the CNIC, MG or cochlea and no apparent gradient of ChAT or GAD+ neurons in the VNTB of control or VPA-exposed neurons. All counts were made in ImageJ (Schindelin et al., 2012) using the cell counting feature by an observer blind to experimental condition. Counts were combined for each animal; the analyses are based on combined proportions of retrogradely labeled neurons in each nucleus.

### Statistical Analysis

Descriptive statistics were generated for each control and VPA group using GraphPad Prism 7.03 (GraphPad Software, La Jolla, CA, United States). All data sets were tested against a normal distribution using the D’Agostino and Pearson omnibus normality test. If a data set was too small for normality testing, non-parametric tests were used (i.e., Mann–Whitney *U* test) and data are presented in the text as median with the 95% confidence interval (CI) of the median. The proportions of IL and CL labeled neurons were compared using Fisher’s exact test. Differences were considered statistically significant if *p*-values were <0.05.

## Results

Features of the Ventral Nucleus of the Trapezoid Body

The VNTB is situated within the SOC along the ventral aspect of the pons amongst the decussating axons of the trapezoid body (Figure 2). The VNTB extends rostrocaudally along nearly the entire extent of the SOC (Figures 2A1–4). Consistent with smaller brains and brainstems in VPA-exposed animals (Zimmerman et al., 2018; Mansour et al., 2019), the SOC is shorter in the rostrocaudal dimension, the constituent nuclei contain significantly fewer neurons and surviving neurons exhibit dysmorphology (Zimmerman et al., 2018). Specifically, in control animals the SOC extends a rostrocaudal distance of 1,457 ± 142 μm and in VPA-exposed animals this is significantly reduced to 1,160 ± 98 μm [*t*(11) = 4.3, *p* = 0.0012]. Consistent with this shortened rostrocaudal distance, the VNTB extends a significantly shorter distance in VPA exposed animals {control = 1,371 ± 98, VPA = 1,040 ± 98 μm; [*t*(11) = 5.22, *p* = 0.0003]}. Despite the significant change in rostrocaudal length and drastic changes in the surrounding SOC nuclei, the VNTB exhibits no significant changes in total nuclear volume, neuron number, or neuronal morphology along its rostrocaudal dimension (Zimmerman et al., 2018; Figures 2B1–4).

### Ascending Projections

**FIGURE 3:**
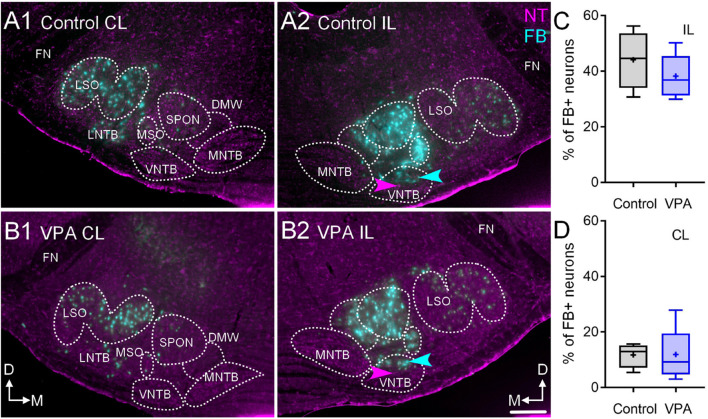
Retrograde labeling after FB injection in the CNIC. Shown in **(A1–2)** are sections through the SOC after FB injection in the CNIC of a control animal (**A1**, CL and **A2**, IL) and **(B)** shows sections from a VPA-exposed animal (**B1**, CL and **B2**, IL). While there are fewer FB+ neurons in the VPA-exposed animal, there was no difference in the number of FB+ neurons in the VNTB IL or CL to the injection. The proportions of FB+ neurons IL to the injection are shown in **(C)** and those CL to the injection are shown in **(D)**. The scale bar in **(B2)** is equal to 100 μm.

After injections of FB in the right CNIC, we found that in control animals 47% (CI 31–56%) of neurons in the IL VNTB and 12.95% (CI 6–17%) of neurons in the CL VNTB were FB+ (Figures 3A1,A2,C,D). In VPA-exposed animals, we found that 37% (CI 30–50%) of neurons in the IL VNTB and 9.28% (CI 3–28%) of neurons in the CL VNTB were FB+ (Figures 3B1,B2,C,D). These differences were not significant [CL: *U*(4,6) = 11, *p* = 0.91; IL: *U*(4,6) = 8, *p* = 0.47] (Figures 3C,D). The difference in proportions of CL/IL projections from the VNTB was similar between control and VPA-exposed animals (Fisher’s exact, *p* > 0.99).

**FIGURE 4:**
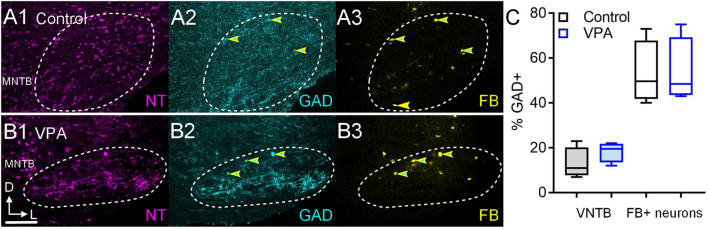
GAD+ VNTB neurons projecting to the CNIC. Shown in **(A1–3)** are sections through the VNTB after a FB injection in the CNIC of a control animal and similar sections are shown from a VPA-exposed animal in **(B1–3)**. GAD immunolabeling is shown in cyan (2) and FB is shown in yellow (3). Neurons that are both GAD and FB+ are indicated by the yellow and cyan arrowheads. The scale bar in **(B2)** is equal to 250 μm. The percentage of GAD+ VNTB neurons is shown in **(C left)** and the percentage of neurons that were GAD and FB+ are shown in **(C right)**.

We also examined the number of VNTB neurons that were GABAergic and the number of FB+/GAD+ after injections in the IL CNIC (Figure 4). In control animals 11% (CI 7–23%) of VNTB neurons were GAD+ and in VPA-exposed animals 19% (CI 12–22%) of VNTB neurons were GAD+ and this was not significant [*U*(4,4) = 4.5, *p* = 0.37; Figure 4C]. After injection of FB in the IL CNIC, 50% (CI 40–73%) of FB+ neurons in the VNTB were GAD+ (Figures 4A1–3). In VPA-exposed animals, 49% (CI 43–75%) of FB+ neurons in the VNTB were GAD+ (Figures 4B1–3). This difference was not significant [*U*(4,4) = 8, *p* > 0.99; Figure 4C]. In both control and VPA-exposed animals, none of the neurons retrogradely labeled from injection of FB in the CNIC were ChAT+ (0/26 control; 0/19 VPA).

**FIGURE 5:**
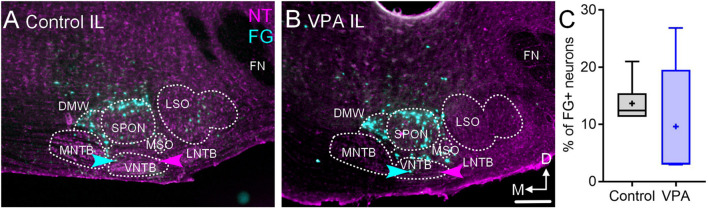
Retrograde labeling after FG injection in the MG. Shown in **(A)** is a section through the IL SOC after FG injection in the MG of a control animal and **(B)** shows a section from a VPA-exposed animal. While there are fewer FG+ neurons in the SOC of VPA-exposed animals, there was no difference in the number of FG+ neurons in the VNTB IL to the injection. The proportions of FG+ neurons IL to the injection are shown in **C**. The scale bar in B2 is equal to 100 μm.

After injections of FG in the right MG, 12% (CI 11–21%) of neurons in the IL VNTB were FG+ in control animals and 10% (CI 3–26%) were FG+ in VPA-exposed animals (Figures 5A–C). This difference was not significant [*U*(6,5) = 9, *p* = 0.30; Figure 5C]. In both control and VPA-exposed animals less than 1% of neurons in the CL VNTB were FG+.

### Cochlea Injections

**FIGURE 6:**
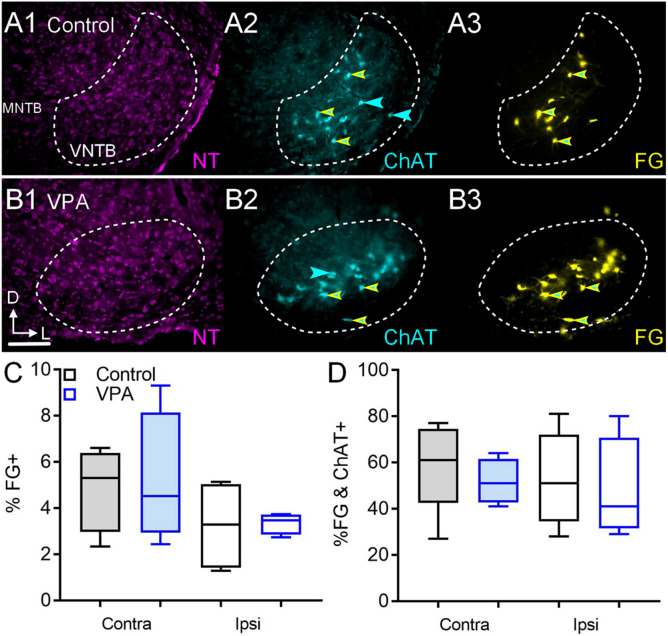
Retrograde labeling after FG injection in the cochlea. Shown in **(A1–3)** are sections through the VNTB after FG injection in the cochlea of a control animal with immunolabeling for ChAT (2). **(B1–3)** shows the VNTB from a VPA-exposed animal. The percentage of FG+ neurons IL and CL to the injection are shown in **(C)**. The percentage of VNTB neurons that were FG and ChAT+ after a cochlear injection are shown in **(D)**. The scale bar in **(B1)** is equal to 250 μm.

After FG deposits through the right round window in control animals, 5.31% (CI 2–7%) of VNTB neurons CL to the injection were FG+ and 3.2% (CI 1–5%) were FG+ IL to the injection (Figure 6C). After similar injections in VPA-exposed animals, 4.5% (CI 2–9%) of VNTB neurons CL to the injection were FG+ and 3.4% (CI 3–4%) were FG+ IL to the injection (Figure 6C). Neither of these differences were significant [CL: *U*(4,4) = 3, *p* = 0.20; IL: *U*(4,4) = 4, *p* = 0.34]. The ratio of IL/CL FG+ neurons was 0.60 in control and 0.75 in VPA-exposed animals. The difference in proportions of CL/IL projections from the VNTB to the cochlea was similar between control and VPA-exposed animals (Fisher’s exact, *p* > 0.99).

In control animals, 5% (CI 2–8%) of VNTB neurons were ChAT+ and 7.6% (CI 2–9%) of VNTB neurons were ChAT+ in VPA-exposed animals. This difference was not significant [*U*(6,7) = 10, *p* = 0.13]. CL to the cochlear injection, we found that in control animals 61% (CI 27–77%) of FG+ neurons in the VNTB were ChAT+ (double labeled; Figures 6A1–3). In VPA-exposed animals 51% (CI 41–64%) of FG+ neurons in the VNTB were ChAT+ (Figures 6B1–3). This difference was not significant [*U*(5,4) = 6, *p* = 0.41; Figure 6D]. IL to the cochlear injection, 51% (CI 28–81%) of FG+ neurons in the VNTB were ChAT+ (double labeled) in control animals. In VPA-exposed animals 41% (CI 29–80%) of FG+ neurons in the VNTB were ChAT+. This difference was not significant [*U*(5,4) = 8, *p* = 0.73; Figure 6D].

## Discussion

**FIGURE 7:**
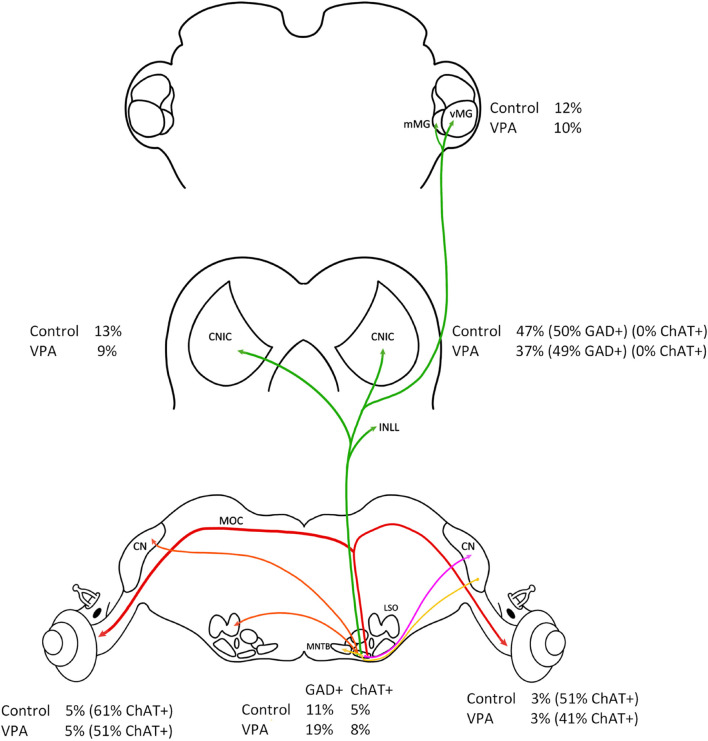
Summary of results. This figure shows a summary of all results presented in this study. All values are relative to the right (IL) VNTB. There was no difference in the percentage of VNTB neurons projecting to the IL or CL cochlea (red line) and no difference in the number of ChAT+ neurons in the VNTB. There was also no difference in the percentage of neurons projecting to the CNIC and MG (green line) and no difference in the number of GAD+ neurons.

This study was motivated by the observations that the VNTB was preserved in both human subjects with ASD and VPA-exposed animals despite well documented auditory processing issues in ASD and significantly hypotrophy and dysmorphology throughout the auditory brainstem in ASD and VPA-exposed animals. Our initial observations that the VNTB was unaffected in these conditions was intriguing since VNTB neurons share a number of developmental features with other SOC nuclei, including origin, lineage and birthday (Altman and Bayer, 1980; Maricich et al., 2009; Marrs et al., 2013). It is important to emphasize that the VNTB is a heterogeneous nucleus in neuronal morphologies, functions and projections but also by origin. Specifically, the majority of VNTB neurons are derived from rhombomeres 3 and 5 while MOC neurons are unique in the SOC in their origin from rhombomere 4 (Di Bonito et al., 2013; Marrs et al., 2013; Altieri et al., 2016). Regardless, our previous work showed that in VPA-exposed animals the VNTB had the same total number of neurons, and these neurons had the same cell body size and shape (Zimmerman et al., 2018). Specifically, there was no difference in the proportions of neuronal morphologies in the VNTB between control and VPA-exposed animals (62–69% round/oval neurons, 18–26% stellate, and 12–13% fusiform; Zimmerman et al., 2018). Furthermore, there was no difference in cell body size in VNTB neurons between control and VPA-exposed animals even when split by cell body shape (Zimmerman et al., 2018). The results presented in this report show there is no significant difference in the proportion of VNTB neurons projecting to the CNIC, MG, and cochlea (Figure 7). Transport of FG from the injection site requires uptake and retrograde transport from axon terminals to the cell body. We worked with the assumption that these processes are normal in VPA-exposed animals. Additionally, we recognize that our tract tracing paradigms may not label all VNTB neurons projecting to these targets, but we expected to label the vast majority of these neurons. We also found no change in the number of VNTB neurons that were GAD+ or ChAT+ and there were no changes in the number of retrogradely labeled VNTB neurons that were GAD+ or ChAT+ (Figure 7). It is possible that some GAD or ChAT immunonegative neurons contain levels of these proteins below the detection levels of our imaging equipment. Further, it is possible our counting paradigm under sampled neuron profiles – however this would have affected counts from both control and VPA-exposed animals. After cochlear injections of FG, we found a number of ChAT+ neurons that were FG negative – these likely represent neurons projecting to the CL cochlea. Based on these observations, we still have no evidence that the VNTB is impacted in ASD or in our animal model of ASD. Below we review the impact of VPA exposure on the SOC and discuss possible features of the VNTB that may subserve its protection in these conditions.

We have examined the SOC in 43 subjects diagnosed with ASD ranging in age from 2 to 56 years of age (Kulesza and Mangunay, 2008; Kulesza et al., 2011; Lukose et al., 2015) and we recently constructed 3D models of the SOC nuclei from seven subjects with ASD (2–11 years of age; Mansour and Kulesza, 2020). We found significant changes in the number of neurons in the surrounding SOC nuclei and drastic dysmorphology in the MSO. However, we found no differences in the volume or number of neurons in the VNTB and there were no changes in neuron size or morphology in subjects with ASD. Additionally, we have studied the structure and connectivity of the SOC in animals exposed to VPA *in utero*. After VPA exposure, we find significantly fewer neurons in all SOC nuclei except the LNTB and VNTB and we find significantly smaller neurons in all SOC nuclei except the VNTB (Zimmerman et al., 2018). Furthermore, there was no change in the proportions of stellate or fusiform neurons in the VNTB (Zimmerman et al., 2018). It is important to note that efferent innervation of outer hair cells in human is relatively sparse and attenuates with age (Liberman and Liberman, 2019). Accordingly, the contribution of the different VNTB subpopulations to the repertoire of VNTB functions may vary from rodent to humans. Besides changes in neuron number and morphology, VPA exposure results in abnormal tonotopic maps (Dubiel and Kulesza, 2016), reduced immunolabeling for the calcium binding proteins calbindin in octopus cells in the VCN, MNTB, and dorsal nucleus of the lateral lemniscus (DNLL; Zimmerman et al., 2018; Mansour et al., 2019) and calretinin in globular bushy cells and calyceal axons (Zimmerman et al., 2018). VPA exposure also results in significantly smaller axon diameters in the trapezoid body and lateral lemniscus (Zimmerman et al., 2018; Mansour et al., 2019). Recently we have examined the impact of VPA exposure on ascending projections to the midbrain and thalamus. VPA exposure resulted in not only fewer neurons in the LSO, MSO, and SPON but also a lower proportion of these neurons in these nuclei were retrogradely labeled from injections of FB in the CNIC (Zimmerman et al., 2020). In fact, the MSO projection was the most severely impacted among the ascending projections to the CNIC. Further, VPA exposure resulted in 31% fewer CNIC neurons (Mansour et al., 2019), 50% fewer neurons in the ventral nucleus of the medial geniculate (vMG) and 55% fewer neurons in the medial nucleus of the medial geniculate (mMG; Mansour et al., 2021). Like the ascending projections to the CNIC, VPA exposure resulted in significantly reduced projections to the MG from the CN, SOC, and CNIC (Mansour et al., 2021). In the SOC, we found both overall and proportionally reduced projections to the MG from the IL LSO, MSO, SPON, and dorsal medial wedge (DMW). In both of these retrograde tracing studies, we found not only reduced projections but abnormal patterns of inputs from the SOC and VCN to the CNIC and MG (see figure 11 in Mansour et al., 2021). The current study provides data showing that the major ascending projections to the CNIC and MG and descending projections to the cochlea from the VNTB are not impacted by VPA exposure. Not only does VPA not impact the projections of the VNTB, but it also does not appear to impact the neurotransmitter profile of these neurons (Figure 7). We found significantly fewer neurons in the CNIC and MG after VPA exposure, but the VNTB projections were normal. It is unclear how VNTB axons terminate in these locations. Specifically, does a single VNTB axon contact more neurons or spread across larger territories in the CNIC and MG of VPA-exposed animals? We will attempt to investigate this question with a combination of anterograde tract tracing and immunohistochemistry. The circuitry of the VNTB is complex and it is important to recognize that we have not examined all projections. Small, focal injections of tracers into the SOC and cochlear nuclei would be required to study these connections, but we hypothesize that these connections are unaffected as well. It is important to note that we have utilized morphometric techniques, immunohistochemistry, and tract tracing strategies to study the VNTB. We have not directly examined function of these neurons and it is possible that sound-evoked responses of VNTB neurons are impaired and/or that function of these neurons is disrupted by abnormal features of target cells in the cochlea, IC, and MG. Notwithstanding, the reason the VNTB is preserved in ASD and in VPA exposures is unclear. However, given these are neurodevelopmental conditions, we propose a mechanism related to developmental origins and protein expression.

The VNTB, along with the MNTB, LNTB, and SPON are most likely derived from the basal plate between E12 and 16 and among the SOC nuclei, have a unique expression pattern of transcription factors (Altman and Bayer, 1980; Kudo et al., 2000; Marrs et al., 2013). Specifically, about 80% of VNTB neurons express *En1* with smaller populations that express *FoxP1* and co-express these markers (Marrs et al., 2013). This pattern is not found in any of the other SOC nuclei but is most closely matched by the LNTB, but only about 45% of LNTB neurons express *En1* (Marrs et al., 2013). Our VPA exposure occurs between E10 and 12, primordial SOC neurons express *En1* as early as E12.5 and the VNTB, MNTB, and LNTB appear to express *En1* until at least P10 (Marrs et al., 2013). *En1* is mainly expressed during development, promotes cell survival through a mitochondrial cascade and protects neurons against cell death (Beltran et al., 2013). *En2* has been implicated in ASD and in the hindbrain this gene is primarily expressed by monoaminergic neurons projecting to the forebrain (Genestine et al., 2015). Dopaminergic neurons in the midbrain substantia nigra express *En1* and in animals heterozygous for *En1* more of these neurons show pathological changes and progressively degenerate (Simon et al., 2001; Alberi et al., 2004; Sonnier et al., 2007; Chatterjee et al., 2019). Furthermore, infusion of engrailed protein into the midbrain protects dopaminergic neurons in the substantia nigra from cell death in animal models of exposure-based Parkinson’s disease (Chatterjee et al., 2019). Interestingly, *En1* is also overexpressed in aggressive forms of breast cancer (Beltran et al., 2013). It would appear then that *En1* plays an important role in cell survival. In mouse models with an *En1* deletion, the MNTB, VNTB do not form and no GABA/glycinergic neurons form in the ventral nucleus of the lateral lemniscus (VNLL; Jalabi et al., 2013; Altieri et al., 2016). As such, it appears that *En1* expression is essential for development of the vast majority of VNTB neurons. Given that nearly all VNTB neurons express *En1* (except MOC neurons derived from rhombomere 4), we interpret our results to suggest this transcription factor, and/or signaling cascades downstream of *En1*, serves to protect the non-MOC VNTB neurons from the *in utero* effects of VPA and neuropathological sequelae of ASD. Since non-MOC VNTB neurons do not form in *En1* knockout animals (Altieri et al., 2016), it would be difficult to examine the impact of *in utero* VPA exposure on this nucleus. However, we hypothesize that VPA exposure in *En1* deficient/heterozygous animals would have much more drastic effects. It is unclear if the VNTB is intact in *En1* ± animals, but if it is, we suspect VPA exposure would result in significantly fewer neurons and dysmorphology. Since MOC neurons in the VNTB are not derived from the *En1* lineage, some other transcription factors or mechanism must protect these neurons (Di Bonito et al., 2013; Marrs et al., 2013; Altieri et al., 2016). Our results suggests that less than 10% of VNTB neurons are ChAT+ and so MOC neurons are a minor component of the VNTB. Our study of brainstem oropharyngeal motor neurons in VPA-exposed animals revealed no changes in the total number of neurons in the facial nucleus, glossopharyngeal nucleus, trigeminal nucleus, or nucleus ambiguous (Alhelo and Kulesza, 2021) suggesting their motor/cholinergic lineage provides protection against premature cell death by *in utero* VPA exposure. Additionally, this minor populations of VNTB neurons may be protected through the local milieu and involve local signaling. VPA, through a number of mechanisms, increases GABA levels in the brain but also acts as a histone deacetylase inhibitor, through which it impacts expression of numerous genes (Göttlicher, 2004). It is unclear what role elevated GABA levels might play in the SOC and VNTB at E10 and E12.5, although GABA receptors are present as early as E11.5 in cortical neurons (Li et al., 2006).

The protective role of *En1* for the VNTB in ASD/VPA exposure is complicated by the fact that other SOC neurons express *En1* (Marrs et al., 2013). Nearly 50% of LNTB neurons express *En1* but ∼90% of MNTB neurons express *En1* and *FoxP1* (Marrs et al., 2013). Again, it may be signaling pathways downstream of engrailed that protect VNTB neurons and/or expression in targets of VNTB axons. Additionally, the timeframe for *En1* expression in the VNTB is unclear. Since the vast majority of VNTB neurons express *En1* it seems these neuronal subtypes (with the exception of MOC neurons) share the same *En1*/engrailed protection. Regardless, our results provide evidence that neurons derived from certain neuronal lineages may be less susceptible to the effects of neurodevelopmental or neurodegenerative conditions and serve as an important foundation into such protective mechanisms.

## Data Availability Statement

The raw data supporting the conclusions of this article will be made available by the authors, without undue reservation.

## Ethics Statement

The animal study was reviewed and approved by the LECOM Institutional Animal Care and Use Committee.

## Author Contributions

YM designed the study, performed the experiments, collected and analyzed the data, created the figures, and edited and approved the manuscript. RK designed the study, provided the resources, performed the experiments, analyzed the data, created the figures, drafted the manuscript, edited and approved the manuscript. Both authors contributed to the article and approved the submitted version.

## Conflict of Interest

The authors declare that the research was conducted in the absence of any commercial or financial relationships that could be construed as a potential conflict of interest.

## Publisher’s Note

All claims expressed in this article are solely those of the authors and do not necessarily represent those of their affiliated organizations, or those of the publisher, the editors and the reviewers. Any product that may be evaluated in this article, or claim that may be made by its manufacturer, is not guaranteed or endorsed by the publisher.

## Abbreviations

+: positive
AN: auditory nerve
ASD: autism spectrum disorder
ChAT: choline acetyltransferase
CI: confidence interval
CL: contralateral
CN: cochlear nucleus
CNIC: central nucleus of the inferior colliculus
D: dorsal
DMW: dorsal medial wedge
DNLL: dorsal nucleus of the lateral lemniscus
E: embryonic
FB: Fast Blue
FG: Fluorogold
FN: facial nerve
GABA: gamma amino butyric acid
GAD: glutamate decarboxylase
IL: ipsilateral
LNTB: lateral nucleus of the trapezoid body
LSO: lateral superior olive
M: medial
MG: medial geniculate
mMG: medial nucleus of the medial geniculate
MNTB: medial nucleus of the trapezoid body
MSO: medial superior olive
NHS: normal horse serum
nl: nanoliter
NTR: Neurotrace Red
OC: olivocochlear
OCB: olivocochlear bundle
P: postnatal
PBS: phosphate buffered saline
PFA: paraformaldehyde
SC: superior colliculus
SOC: superior olivary complex
SPON: superior paraolivary nucleus
tz: trapezoid body
VCN: ventral cochlear nucleus
vMG: ventral nucleus of the medial geniculate
VNLL: ventral nucleus of the lateral lemniscus
VNTB: ventral nucleus of the trapezoid body
VPA: valproic acid.
